# Re-investigation and correct symmetry of Ca_3_CoAl_4_O_10_

**DOI:** 10.1107/S2056989019000574

**Published:** 2019-01-15

**Authors:** Volker Kahlenberg

**Affiliations:** a University of Innsbruck, Institute of Mineralogy & Petrography, Innrain 52, A-6020 Innsbruck, Austria

**Keywords:** crystal structure, Ca_3_CoAl_4_O_10_, redetermination, symmetry analysis

## Abstract

A re-investigation of the crystal structure of tricalcium cobalt(II) tetra­aluminate, Ca_3_CoAl_4_O_10_, using single-crystal X-ray diffraction data, revealed ortho­rhom­bic (*Pbcm*) symmetry. The present contribution corrects the results of a previous X-ray powder diffraction study [Vazquez *et al.* (2002 ▸). *J. Solid State Chem.* **166**, 191–196] where this phase has been described in an unnecessarily low space-group symmetry (*Pbc*2_1_). The compound belongs to the group of tetra­hedral framework structures. The distribution of the aluminium and cobalt ions among the centres of the four different tetra­hedra within the asymmetric unit has been studied in detail. Charge compensation is achieved by the incorporation of two symmetrically independent calcium ions located in voids of the tetra­hedral framework. Ca_3_CoAl_4_O_10_ is isotypic with Ca_3_MgAl_4_O_10_.

## Abstract 2

The crystal structure of Ca_3_CoAl_4_O_10_ was redetermined from single-crystal X-ray data and is isotypic with Ca_3_MgAl_4_O_10_.

## Chemical context   

In a recent paper on the phase relationships in the system CaO–MgO–Al_2_O_3_, we reported the existence and the crystal structure of Ca_3_MgAl_4_O_10_ (Kahlenberg *et al.*, 2018 ▸), a phase of inter­est for slags occurring in secondary refining processes in metallurgy or refractories, for example. In the course of this study it became obvious that the compound is closely related to the corresponding Zn and Co analogues that have already been reported in the literature (Barbanyagre *et al.*, 1997 ▸; Vazquez *et al.*, 2002 ▸). In fact Vazquez *et al.* (2002 ▸) used the coordinates from the Zn compound as a starting model for their Rietveld refinement of Ca_3_CoAl_4_O_10_. The major difference from our investigation on Ca_3_MgAl_4_O_10_ results from the fact that the previous study attributed Ca_3_CoAl_4_O_10_ to the acentric space group *Pbc*2_1_, while Ca_3_MgAl_4_O_10_ crystallizes in the centrosymmetric space group *Pbcm*. However, for the former compound the description in an acentric space group has to be scrutinized. A detailed analysis of the atomic coordinates using the program *PSEUDO* (Kroumova *et al.*, 2001 ▸) indicated that the published model fulfills the symmetry requirements of *Pbcm*. Notably, Vazquez *et al.* (2002 ▸) reported problems during their structure analysis of Ca_3_CoAl_4_O_10_, including unstable refinements and unrealistically short cation–oxygen distances. Both observations are typical features when a structure is refined in an unnecessarily low space-group symmetry (Baur & Tillmanns, 1986 ▸). Therefore, it was deemed appropriate to re-investigate the crystal structure of Ca_3_CoAl_4_O_10_ using single-crystal X-ray diffraction data obtained from melt-grown crystals.

## Structural commentary   

**Figure 1:**
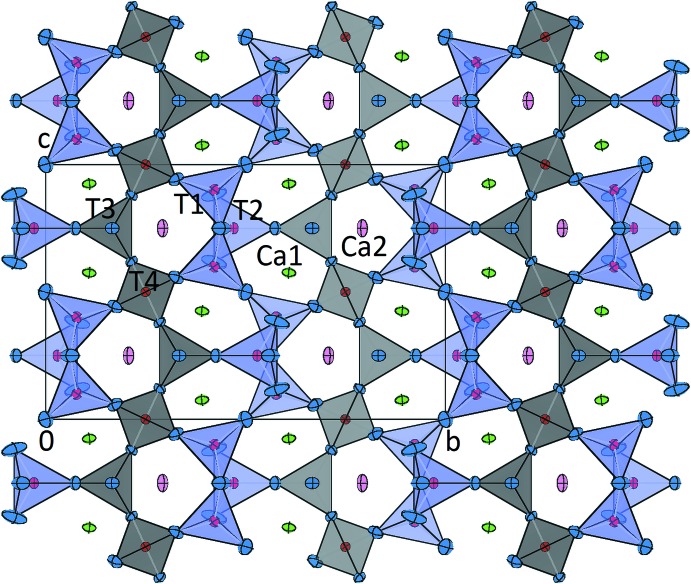
Projection of the crystal structure of Ca_3_CoAl_4_O_10_ in a view parallel to [100]. Displacement ellipsoids are shown at the 90% probability level. Colour codings for the atoms and polyhedra are as follows: blue: oxygen; red: aluminium/cobalt; Ca1: pink; Ca2: green; cobalt-containing tetra­hedra ([*T*1O_4_] and [*T*2O_4_]): lilac; pure aluminium tetra­hedra ([*T*3O_4_] and [*T*4O_4_]): grey.

**Figure 2:**
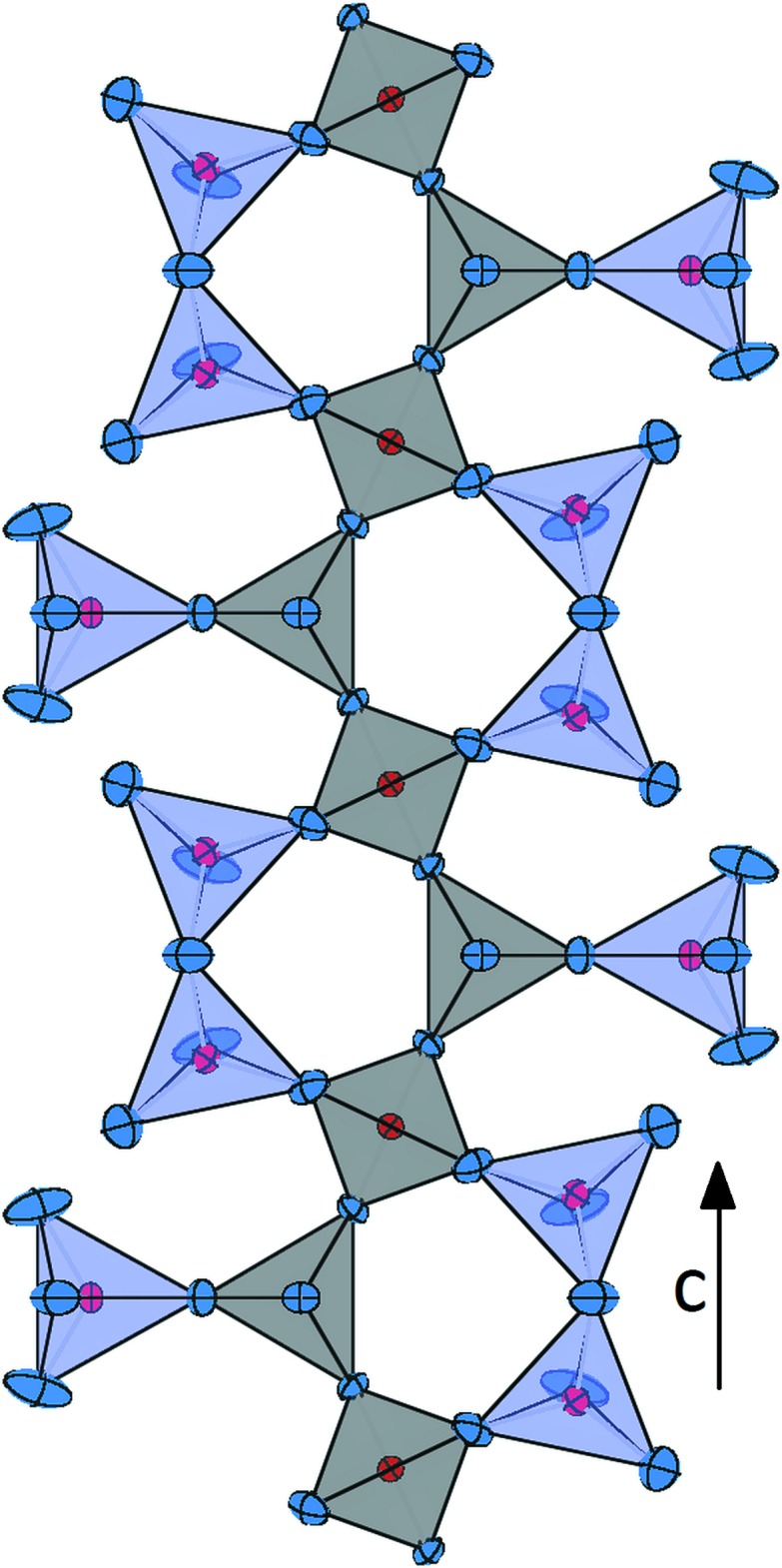
A single mixed-branched *vierer* single chain (running parallel to [001]) representing the backbone of the framework in Ca_3_CoAl_4_O_10_. Displacement ellipsoids as in Fig. 1 ▸.

**Figure 3:**
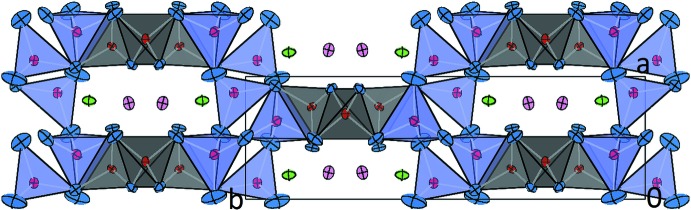
Projection of the crystal structure of Ca_3_CoAl_4_O_10_ in a view parallel to [001]. Displacement ellipsoids as in Fig. 1 ▸.

The crystal structure of Ca_3_CoAl_4_O_10_ can be described as a three-dimensional network with four symmetrically different corner-sharing [(Al,Co)O_4_] tetra­hedra around the central atoms *T*1–*T*4 (Fig. 1 ▸). The basic building units of the structure are chains of tetra­hedra running parallel to [001]. Using the crystal chemical classification developed by Liebau (1985 ▸), these linear elements can be described as mixed-branched *vierer* single chains (Fig. 2 ▸). Condensation of adjacent chains along [010] results in the formation of stepped layers parallel to (100) (Fig. 3 ▸). Within these layers, channels can be identified which host the additional calcium ions.

Site-occupancy refinements indicated that cobalt incorporation is limited to two of the four *T* sites within the asymmetric unit (*T*1 and *T*2). *T*3 and *T*4 are virtually cobalt free. The spread of the individual *T*—O bond lengths and the O—*T*—O angles follow expected crystallochemical trends. For the average *T*—O values, two groups can be distinguished: *T*1, *T*2: 1.808 Å and *T*3, *T*4: 1.758 Å. These values reflect the larger ionic radius of Co^2+^ for fourfold coordination [*r*(Co^2+,[4]^): 0.58 Å] when compared to the corresponding value for Al^3+^ [*r*(Al^3+,[4]^): 0.39 Å] (Shannon, 1976 ▸), and can be used as an indication that *T*1 and *T*2 have higher Co contents. This observation compares well with the site-population refinements. Quadratic elongations as defined by Robinson *et al.* (1971 ▸), which can be used as numerical descriptors for the distortions, take the following values for the individual [(Al,Co)O_4_]-groups: *T*1: 1.015, *T*2: 1.006, *T*3: 1.016, *T*4: 1.001.

Among the extra-framework cations, two crystallographically independent calcium sites (Ca1, Ca2) can be distinguished. They are coordinated by six and eight nearest oxygen neighbours. Their coordination polyhedra can be described as distorted octa­hedra and square anti­prisms, respectively. Each two [Ca1O_6_] octa­hedra and a single [Ca2O_8_] square anti­prism form a polyhedral unit by sharing edges.

A detailed analysis of the topological features of the tetra­hedral network including coordination sequences and extended point symbols has been already presented for isotypic Ca_3_MgAl_4_O_10_ (Kahlenberg *et al.*, 2018 ▸) and will not be duplicated here. However, it is inter­esting to note that the framework consists of three (*T*3), four (*T*4) and five (*T*1, *T*2)-connected tetra­hedra. Notably, the net contains an O^[3]^-type bridging oxygen (O3), simultaneously linking three tetra­hedra (one [*T*2O_4_]- and two [*T*1O_4_]-units). In oxo-silicates that are based on [SiO_4_] units, for example, only terminal (O^[1]^) and simple bridging (O^[2]^) oxygen atoms have been observed so far. In the present structure, the oxygen atoms O1 and O2, O4, O5, O6, O7 belong to these two groups. Notably, O3 is solely involved in O—*T* bonds with the two tetra­hedra showing an Al/Co substitution.

## Database survey   

As mentioned above, the title compound is isotypic with Ca_3_MgAl_4_O_10_. For the calculation of several qu­anti­tative descriptors for the characterization of the degree of similarity between the crystal structures of Ca_3_CoAl_4_O_10_ and Ca_3_MgAl_4_O_10_, the program *COMPSTRU* (de la Flor *et al.*, 2016 ▸) was employed. For the given two structures, the degree of lattice distortion *S*, *i.e.* the spontaneous strain obtained from the eigenvalues of the finite Lagrangian strain tensor calculated in a Cartesian reference system, has a value of *S* = 0.0010. The structure of Ca_3_CoAl_4_O_10_ was transformed to the most similar configuration of Ca_3_MgAl_4_O_10_. The calculations revealed the following atomic displacements (in Å) between the corresponding atoms in Ca_3_CoAl_4_O_10_ and Ca_3_MgAl_4_O_10_: Ca1: 0.036; Ca2: 0.028; *T*1: 0.044; *T*2: 0.010; *T*3: 0.027; *T*4: 0.031; O1: 0.026; O2: 0.030; O3: 0.019; O4: 0.024; O5: 0.000; O6: 0.031; O7: 0.040, *i.e.* the maximum displacement is lower than 0.05 Å. The measure of similarity (Δ) as defined by Bergerhoff *et al.* (1999 ▸) has a value of 0.007. Notably, for both structures the divalent cations Co^2+^ and Mg^2+^ are enriched in the tetra­hedral positions *T*1 and *T*2.

The distribution of the cobalt and aluminium ions on the different *T* sites is another difference between the new centrosymmetric model in *Pbcm* (this work) and the previous acentric model in *Pbc*2_1_ (Vazquez *et al.*, 2002 ▸). Actually, in the latter case five different tetra­hedral positions have to be distinguished. The authors considered four of them to be exclusively occupied with Al while the remaining fifth position was attributed to be a pure cobalt site. This distribution, however, was derived from the crystal-structure refinement of the zinc analog (Barbanyagre *et al.*, 1997 ▸) and not determined by site-occupancy refinements.

Furthermore, the new model in *Pbcm* results in considerably less distorted tetra­hedra. Although soft constraints on the Al—O and Co—O bond lengths had been applied, individual *T*—O distances and O—*T*—O angles in the *Pbc*2_1_ structure model showed a pronounced variation between 1.68 and 2.05 Å and 92.9 and 124.6°, respectively. The corresponding values in the present model are in the ranges from 1.719 (4) to 1.847 (2) Å and from 98.95 (16) to 120.38 (18)°, respectively. Finally, the displacement parameters in *Pbcm* are all well behaved, while the overall isotropic temperature factor for the oxygen atoms reported in the study of Vazquez *et al.* (2002 ▸) takes a physically unrealistic value of *U*_iso_ = 0.001 (2) Å^2^.

## Synthesis and crystallization   

Single crystals of Ca_3_CoAl_4_O_10_ were obtained during a series of synthesis experiments in the system CaO–CoO–Al_2_O_3_. 1.35 g of the educts consisting of CaCO_3_, CoO and Al_2_O_3_ in the molar ratio 14:6:5 were homogenized in an agate mortar, transferred into a platinum crucible and covered with a lid. The container was fired in a resistance-heated furnace from 590 to 1623 K with a ramp of 100 K h^−1^. The target temperature was held for 1 h. Subsequently, the sample was cooled down to 1273 K at a rate of 7.5 K h^−1^ and, finally, the temperature was reduced to 473 K at a rate of 100 K h^−1^. After removal of the crucible, the solidified melt cake was immediately crushed in an agate mortar and transferred to a glass slide under a polarizing binocular. A first inspection revealed the presence of two crystalline phases: larger colourless optically isotropic crystals of Ca_3_Al_2_O_6_ (up to 500 µm in size) and considerably smaller, intensively blue birefringent crystals of Ca_3_CoAl_4_O_10_. A platy fragment of the latter compound showing sharp extinction under crossed polarizers was selected for further structural studies and mounted on the tip of a glass fibre using fingernail hardener as glue.

## Refinement   

Crystal data, data collection and structure refinement details are summarized in Table 1 ▸. Starting parameters for the atomic coordinates were taken from the crystal structure of Ca_3_Al_4_MgO_10_ (Kahlenberg *et al.*, 2018 ▸). Initially, mixed cobalt–aluminium populations were considered for all four *T* sites. However, the resulting values of the site occupancies for *T*3 and *T*4 indicated pure Al populations (within two standard uncertainties each). In the final cycles a restraint was introduced, fixing the total amount of cobalt distributed among the remaining *T*1 and *T*2 sites to four atoms per unit cell.

## Supplementary Material

Crystal structure: contains datablock(s) global, I. DOI: 10.1107/S2056989019000574/wm5480sup1.cif

Structure factors: contains datablock(s) I. DOI: 10.1107/S2056989019000574/wm5480Isup2.hkl

CCDC reference: 1890066

Additional supporting information:  crystallographic information; 3D view; checkCIF report

## Figures and Tables

**Table 1:** Experimental details

Crystal data	
Chemical formula	Al_4_Ca_3_CoO_10_
*M*_r_	447.09
Crystal system, space group	Orthorhombic, *P* *b* *c* *m*
Temperature (K)	293
*a*, *b*, *c* (Å)	5.1324 (6), 16.7550 (19), 10.6822 (12)
*V* (Å^3^)	918.60 (18)
*Z*	4
Radiation type	Mo *K*α
μ (mm^−1^)	3.97
Crystal size (mm)	0.12 × 0.09 × 0.06

Data collection	
Diffractometer	Rigaku Oxford Diffraction Xcalibur, Ruby, Gemini ultra
Absorption correction	Analytical (*CrysAlis PRO*; Rigaku OD, 2015 ▸). Analytical numeric absorption correction using a multifaceted crystal model based on expressions published by Clark & Reid (1995 ▸).
*T*_min_, *T*_max_	0.759, 0.898
No. of measured, independent and observed [*I* > 2σ(*I*)] reflections	5719, 973, 791
*R*_int_	0.065
(sin θ/λ)_max_ (Å^−1^)	0.621

Refinement	
*R*[*F* ^2^ > 2σ(*F* ^2^)], *wR*(*F* ^2^), *S*	0.030, 0.061, 1.07
No. of reflections	973
No. of parameters	96
No. of restraints	1
Δρ_max_, Δρ_min_ (e Å^−3^)	0.6, −0.64

Computer programs: *CrysAlis PRO* (Rigaku OD, 2015 ▸), *SHELXL2017/1* (Sheldrick, 2015 ▸), *VESTA3* (Momma & Izumi, 2011 ▸), *publCIF* (Westrip, 2010 ▸) and *WinGX* (Farrugia, 2012 ▸).

## supplementary crystallographic information

### Crystal data

**Table:**

Ca_3_CoAl_4_O_10_	*F*(000) = 876
*M**_r_* = 447.09	*D*_x_ = 3.233 Mg m^−^^3^
Orthorhombic, *P**b**c**m*	Mo *K*α radiation, λ = 0.71073 Å
Hall symbol: -P 2c 2b	Cell parameters from 939 reflections
*a* = 5.1324 (6) Å	θ = 5.0–28.4°
*b* = 16.7550 (19) Å	µ = 3.97 mm^−^^1^
*c* = 10.6822 (12) Å	*T* = 293 K
*V* = 918.60 (18) Å^3^	Fragment, colourless
*Z* = 4	0.12 × 0.09 × 0.06 mm

### Data collection

**Table:**

Rigaku Oxford Diffraction Xcalibur, Ruby, Gemini ultra diffractometer	973 independent reflections
Radiation source: fine-focus sealed X-ray tube, Enhance (Mo) X-ray Source	791 reflections with *I* > 2σ(*I*)
Graphite monochromator	*R*_int_ = 0.065
Detector resolution: 10.3575 pixels mm^-1^	θ_max_ = 26.2°, θ_min_ = 3.8°
ω scans	*h* = −5→6
Absorption correction: analytical (CrysAlisPro; Rigaku OD, 2015). Analytical numeric absorption correction using a multifaceted crystal model based on expressions published by Clark & Reid (1995).	*k* = −20→15
*T*_min_ = 0.759, *T*_max_ = 0.898	*l* = −12→10
5719 measured reflections	

### Refinement

**Table:**

Refinement on *F*^2^	1 restraint
Least-squares matrix: full	*w* = 1/[σ^2^(*F*_o_^2^) + (0.0197*P*)^2^ + 0.4838*P*] where *P* = (*F*_o_^2^ + 2*F*_c_^2^)/3
*R*[*F*^2^ > 2σ(*F*^2^)] = 0.030	(Δ/σ)_max_ < 0.001
*wR*(*F*^2^) = 0.061	Δρ_max_ = 0.6 e Å^−^^3^
*S* = 1.07	Δρ_min_ = −0.64 e Å^−^^3^
973 reflections	Extinction correction: SHELXL-2017/1 (Sheldrick 2015), Fc^*^=kFc[1+0.001xFc^2^λ^3^/sin(2θ)]^-1/4^
96 parameters	Extinction coefficient: 0.0014 (4)

### Special details

**Table:**

Geometry. All esds (except the esd in the dihedral angle between two l.s. planes) are estimated using the full covariance matrix. The cell esds are taken into account individually in the estimation of esds in distances, angles and torsion angles; correlations between esds in cell parameters are only used when they are defined by crystal symmetry. An approximate (isotropic) treatment of cell esds is used for estimating esds involving l.s. planes.

### Fractional atomic coordinates and isotropic or equivalent isotropic displacement parameters (Å^2^)

**Table:**

	*x*	*y*	*z*	*U*_iso_*/*U*_eq_	Occ. (<1)
Al1	0.63528 (15)	−0.07769 (4)	0.59968 (7)	0.0076 (2)	0.6196 (19)
Co1	0.63528 (15)	−0.07769 (4)	0.59968 (7)	0.0076 (2)	0.3804 (19)
Al2	0.1321 (2)	−0.02939 (7)	0.75	0.0068 (4)	0.761 (4)
Co2	0.1321 (2)	−0.02939 (7)	0.75	0.0068 (4)	0.239 (4)
Al3	0.2463 (3)	0.16550 (9)	0.75	0.0043 (3)	
Al4	0.3045 (3)	0.25	0.5	0.0062 (4)	
Ca1	0.80287 (14)	0.10873 (4)	0.57559 (7)	0.0093 (2)	
Ca2	0.7833 (2)	0.20768 (6)	0.25	0.0131 (3)	
O1	0.5811 (6)	0.1666 (2)	0.75	0.0085 (8)	
O2	0.0758 (7)	0.0742 (2)	0.75	0.0102 (8)	
O3	0.4655 (8)	−0.0615 (2)	0.75	0.0192 (10)	
O4	0.1005 (5)	0.21476 (14)	0.6201 (2)	0.0084 (6)	
O5	0.5	0	0.5	0.0174 (9)	
O6	0.4916 (5)	−0.17398 (15)	0.5570 (2)	0.0130 (6)	
O7	−0.0172 (5)	−0.07854 (19)	0.8828 (2)	0.0249 (7)	

### Atomic displacement parameters (Å^2^)

**Table:**

	*U*^11^	*U*^22^	*U*^33^	*U*^12^	*U*^13^	*U*^23^
Al1	0.0068 (4)	0.0079 (5)	0.0082 (4)	−0.0011 (3)	−0.0006 (3)	−0.0001 (3)
Co1	0.0068 (4)	0.0079 (5)	0.0082 (4)	−0.0011 (3)	−0.0006 (3)	−0.0001 (3)
Al2	0.0072 (7)	0.0052 (7)	0.0080 (7)	0.0001 (5)	0	0
Co2	0.0072 (7)	0.0052 (7)	0.0080 (7)	0.0001 (5)	0	0
Al3	0.0042 (8)	0.0054 (8)	0.0034 (7)	−0.0015 (6)	0	0
Al4	0.0072 (8)	0.0058 (8)	0.0056 (8)	0	0	0.0003 (6)
Ca1	0.0080 (4)	0.0119 (4)	0.0080 (4)	−0.0004 (3)	−0.0010 (3)	−0.0005 (3)
Ca2	0.0116 (6)	0.0077 (6)	0.0200 (6)	−0.0011 (5)	0	0
O1	0.0035 (19)	0.013 (2)	0.0085 (17)	0.0022 (15)	0	0
O2	0.009 (2)	0.010 (2)	0.0120 (18)	0.0015 (15)	0	0
O3	0.021 (2)	0.026 (3)	0.011 (2)	0.0087 (19)	0	0
O4	0.0083 (13)	0.0085 (15)	0.0083 (12)	−0.0018 (10)	−0.0007 (11)	0.0021 (10)
O5	0.017 (2)	0.017 (2)	0.018 (2)	−0.0078 (17)	0.0023 (18)	0.0007 (17)
O6	0.0126 (15)	0.0155 (16)	0.0109 (13)	0.0068 (12)	−0.0013 (11)	−0.0033 (11)
O7	0.0163 (16)	0.043 (2)	0.0152 (14)	−0.0090 (14)	−0.0037 (13)	0.0088 (14)

### Geometric parameters (Å, º)

**Table:**

T1—O7^i^	1.794 (3)	T4—O4^v^	1.758 (2)
T1—O5	1.8194 (7)	Ca1—O6^iv^	2.342 (3)
T1—O6	1.831 (3)	Ca1—O1	2.389 (2)
T1—O3	1.847 (2)	Ca1—O7^vi^	2.389 (3)
T2—O2	1.759 (4)	Ca1—O4^vii^	2.391 (3)
T2—O3	1.794 (4)	Ca1—O2^vii^	2.402 (2)
T2—O7	1.810 (3)	Ca1—O5	2.5273 (8)
T2—O7^ii^	1.810 (3)	Ca2—O1^v^	2.348 (4)
T3—O1	1.719 (4)	Ca2—O4^viii^	2.503 (3)
T3—O2	1.762 (4)	Ca2—O4^ix^	2.503 (3)
T3—O4^ii^	1.780 (3)	Ca2—O6^vi^	2.561 (3)
T3—O4	1.780 (3)	Ca2—O6^iv^	2.561 (3)
T4—O6^iii^	1.758 (3)	Ca2—O3^iv^	2.762 (4)
T4—O6^iv^	1.758 (3)	Ca2—O7^iv^	2.852 (3)
T4—O4	1.758 (2)	Ca2—O7^vi^	2.852 (3)
			
O7^i^—T1—O5	116.51 (11)	O2^vii^—Ca1—O5	115.65 (9)
O7^i^—T1—O6	114.80 (13)	O1^v^—Ca2—O4^viii^	79.73 (9)
O5—T1—O6	109.33 (9)	O1^v^—Ca2—O4^ix^	79.73 (9)
O7^i^—T1—O3	112.30 (14)	O4^viii^—Ca2—O4^ix^	67.34 (11)
O5—T1—O3	102.92 (12)	O1^v^—Ca2—O6^vi^	87.38 (8)
O6—T1—O3	98.95 (16)	O4^viii^—Ca2—O6^vi^	156.79 (9)
O2—T2—O3	116.90 (19)	O4^ix^—Ca2—O6^vi^	91.52 (8)
O2—T2—O7	112.29 (13)	O1^v^—Ca2—O6^iv^	87.38 (8)
O3—T2—O7	105.50 (13)	O4^viii^—Ca2—O6^iv^	91.52 (8)
O2—T2—O7^ii^	112.29 (13)	O4^ix^—Ca2—O6^iv^	156.79 (9)
O3—T2—O7^ii^	105.50 (13)	O6^vi^—Ca2—O6^iv^	107.20 (12)
O7—T2—O7^ii^	103.2 (2)	O1^v^—Ca2—O3^iv^	126.24 (12)
O1—T3—O2	120.38 (18)	O4^viii^—Ca2—O3^iv^	139.56 (7)
O1—T3—O4^ii^	114.55 (12)	O4^ix^—Ca2—O3^iv^	139.56 (7)
O2—T3—O4^ii^	101.18 (12)	O6^vi^—Ca2—O3^iv^	63.24 (7)
O1—T3—O4	114.55 (12)	O6^iv^—Ca2—O3^iv^	63.24 (7)
O2—T3—O4	101.18 (12)	O1^v^—Ca2—O7^iv^	150.06 (6)
O4^ii^—T3—O4	102.48 (17)	O4^viii^—Ca2—O7^iv^	113.33 (9)
O6^iii^—T4—O6^iv^	106.90 (19)	O4^ix^—Ca2—O7^iv^	81.05 (8)
O6^iii^—T4—O4	110.19 (11)	O6^vi^—Ca2—O7^iv^	70.38 (8)
O6^iv^—T4—O4	111.36 (12)	O6^iv^—Ca2—O7^iv^	117.70 (9)
O6^iii^—T4—O4^v^	111.36 (12)	O3^iv^—Ca2—O7^iv^	61.43 (9)
O6^iv^—T4—O4^v^	110.19 (11)	O1^v^—Ca2—O7^vi^	150.06 (6)
O4—T4—O4^v^	106.90 (18)	O4^viii^—Ca2—O7^vi^	81.05 (8)
O6^iv^—Ca1—O1	88.56 (9)	O4^ix^—Ca2—O7^vi^	113.33 (9)
O6^iv^—Ca1—O7^vi^	82.78 (10)	O6^vi^—Ca2—O7^vi^	117.70 (9)
O1—Ca1—O7^vi^	167.86 (11)	O6^iv^—Ca2—O7^vi^	70.38 (8)
O6^iv^—Ca1—O4^vii^	100.70 (9)	O3^iv^—Ca2—O7^vi^	61.43 (9)
O1—Ca1—O4^vii^	81.24 (10)	O7^iv^—Ca2—O7^vi^	59.64 (11)
O7^vi^—Ca1—O4^vii^	91.99 (9)	T2—O2—T3	140.8 (2)
O6^iv^—Ca1—O2^vii^	163.33 (10)	T2—O3—T1^ii^	119.61 (11)
O1—Ca1—O2^vii^	76.74 (10)	T2—O3—T1	119.61 (11)
O7^vi^—Ca1—O2^vii^	110.44 (10)	T1^ii^—O3—T1	120.8 (2)
O4^vii^—Ca1—O2^vii^	69.64 (11)	T4—O4—T3	118.32 (15)
O6^iv^—Ca1—O5	75.31 (7)	T1—O5—T1^iv^	180.00 (4)
O1—Ca1—O5	104.40 (9)	T4^iv^—O6—T1	119.00 (15)
O7^vi^—Ca1—O5	81.67 (7)	T1^x^—O7—T2	119.94 (16)
O4^vii^—Ca1—O5	172.84 (7)		

Symmetry codes: (i) *x*+1, *y*, −*z*+3/2; (ii) *x*, *y*, −*z*+3/2; (iii) −*x*+1, *y*+1/2, *z*; (iv) −*x*+1, −*y*, −*z*+1; (v) *x*, −*y*+1/2, −*z*+1; (vi) −*x*+1, −*y*, *z*−1/2; (vii) *x*+1, *y*, *z*; (viii) *x*+1, −*y*+1/2, −*z*+1; (ix) *x*+1, −*y*+1/2, *z*−1/2; (x) *x*−1, *y*, −*z*+3/2.
